# Association of Depression With Functional Mobility in Schizophrenia

**DOI:** 10.3389/fpsyt.2020.00854

**Published:** 2020-08-21

**Authors:** Jiheon Kim, Ji-Hyeon Shin, Jeh-Kwang Ryu, Jae Hoon Jung, Chan-Hyung Kim, Hwa-Bock Lee, Do Hoon Kim, Sang-Kyu Lee, Daeyoung Roh

**Affiliations:** ^1^Mind-Neuromodulation Laboratory and Department of Psychiatry, Chuncheon Sacred Heart Hospital, Hallym University College of Medicine, Chuncheon, South Korea; ^2^Department of Otolaryngology-Head and Neck Surgery, College of Medicine, The Catholic University of Korea, Uijeongbu, South Korea; ^3^Department of Physical Education, College of Education, Dongguk University, Seoul, South Korea; ^4^Department of Psychiatry, Severance Hospital, Yonsei University College of Medicine, Seoul, South Korea; ^5^Gwangmyeong Mental Health Welfare Center, Gwangmyeong, South Korea

**Keywords:** schizophrenia, depression, functional mobility, pedometer, Timed Up-and-Go test

## Abstract

**Background:**

Functional immobility can cause functional disability in patients with schizophrenia and has been linked to prognosis and mortality. Although depression might be a barrier for physical activity engagement, scarce data are present on the relationship between depression and functional mobility (FM) in schizophrenia. Thus, we aimed to investigate the associations among FM, depression, and other clinical correlates in individuals with schizophrenia.

**Methods:**

FM was evaluated by the pedometer-assessed daily steps and Timed Up-and-Go (TUG) test in the daily-living and clinical settings, respectively. Psychiatric symptoms were assessed using the Beck Depression Inventory, Brief Psychiatric Rating Scale (BPRS), and State-Trait Anxiety Inventory. Cognitive function was evaluated using the Sternberg Working Memory (SWM) Task. Multiple regression analyses were performed to identify predictive factors associated with FM, with adjustment for relevant covariates.

**Results:**

Sixty patients were enrolled in this study. Depression was the most consistent explanatory variable for both pedometer (β = −0.34, p = 0.011) and TUG time (β = 0.32, p = 0.018). Additionally, SWM accuracy (β = −0.29, p = 0.018), BPRS-Withdrawal (β = 0.19, p = 0.139), and fasting blood sugar (β = 0.34, p = 0.008) were associated with TUG time. However, psychotic symptoms and anxiety were not associated with pedometer and TUG.

**Conclusions:**

We identified an association between depression and FM after adjusting for other disorder-related correlates in schizophrenia. Since the intervention goal is functional recovery, improving FM by treating depression may have considerable therapeutic value.

## Introduction

Although psychotic symptoms are a key feature of schizophrenia, the most costly problem in this condition is significant functional disability (1). One of the main causes of functional disability in patients with schizophrenia is impairment in functional mobility (FM) (2). FM is the ability to move from one place to another independently in the environment (3) and requires complex physical processes such as walking, transferring, and turning (4). Because FM enables functional living, including the ability to perform activities of daily living (3), it is necessary to focus on FM in schizophrenia.

Multiple factors may contribute to the FM impairments in patients with schizophrenia, including chronic medical conditions (e.g., diabetes, obesity), sedentary behavior, physical inactivity, or side effects of antipsychotic drugs (5–9). There is also evidence showing that FM predicts overall health decline and disability in activities of daily living (10). Based on these studies, FM can be directly linked to the prognosis and mortality of schizophrenia in the long term. Therefore, in addition to examining the various health factors that may affect FM, it is also important to explore other factors that may affect FM.

One such factor is psychiatric symptoms. A cross-sectional study focusing on psychiatric symptoms found that positive psychotic symptoms were not associated with everyday functioning in patients with schizophrenia (11). In addition, some researchers have shown that the relationship between positive symptoms and FM is not clear (5, 12). These findings imply that patients may experience persistent disability in multiple functional domains even after remission of psychotic symptoms (13). Therefore, it is necessary to examine the effects of other psychiatric symptoms, such as depression, on FM in patients with schizophrenia.

Depressive symptoms in schizophrenia are not only prevalent in the prodromal active and remission phases, but also are militating against long-term recovery (14). Depression results in perceived functional disability and increases the need to utilize healthcare services (15). Furthermore, depression has been found to predict FM limitations (16), and Hirvensalo et al. (17) proposed that depression and FM may progress simultaneously and partially share etiology. Such findings suggest that depression plays an important role in the FM impairments in schizophrenia.

Stubbs et al. (18) have suggested that depressive symptoms might be a barrier for engaging in vigorous physical activity; however, there is still a lack of data on the relationship between depression and FM in schizophrenia. A pedometer or accelerometer can objectively measure physical activity-related mobility without interfering with the normal patterns of life (19, 20). These measurements have previously been applied to assess the mobility level related to physical activity in individuals with mobility limitations (20, 21).

However, such mobility-related activity measurements are limited in their ability to assess the complex physical processes of FM. Therefore, the Timed Up-and-Go (TUG) test (22), a simple, practical, and widely used clinical performance-based test, is often employed to measure FM (4). The psychometric properties of the TUG test have high inter-rater and test-retest reliability (22). Despite its apparent simplicity, the TUG test has the advantage of reflecting multiple components of FM, and thus can help predict the overall functional level of the patient’s daily life (4). To our knowledge, only one prior study has administered the TUG test to patients with schizophrenia (5). In that study, the FM measured by the TUG test was associated with negative symptoms and neurocognition in 46 older adults with schizophrenia, but depressive symptoms were not measured. As such, studies evaluating the relationship between FM, as assessed with the both a pedometer and the TUG test, and depression in schizophrenia are needed.

In the present study, we aimed to investigate the relationships among FM, depression, and other clinical correlates in individuals with schizophrenia. FM was assessed with both a pedometer and the TUG test. The clinical correlates included psychiatric symptoms (depression, anxiety, and psychotic symptoms), psychological variables (quality of life, self-esteem), cognitive function (working memory), and other related clinical factors (diabetes, obesity, and antipsychotic drugs). We hypothesized that lower FM would be associated with higher depressive symptoms, regardless of other clinical correlates, in patients with schizophrenia.

## Methods

### Participants and Design

A cross-sectional design was used to examine the hypothesis. All procedures were approved by the Investigational Review Board of Severance Mental Health Hospital (IRB#: YJI130702). All participants provided written informed consent.

Patients aged 18 to 60 with a primary diagnosis of schizophrenia or schizoaffective disorder were recruited *via* posters placed in local hospitals with psychiatric units and community mental health centers, and *via* advertisements in local newspapers. Some patients were also referred by other local health professionals. Participants were included if they had a diagnosis of schizophrenia or schizoaffective disorder according to the Diagnostic and Statistical Manual for Mental Disorders, 4th Edition, Text Revision. Face-to-face diagnostic interviews were conducted by a psychiatric/mental health nurse and a board-certified psychiatrist who reviewed the history, symptoms, and psychosocial function of each patient using all available sources of information, in accordance with DSM-IV-TR criteria. All participants were required to be on a stable dose and dosing regimen of antipsychotics for at least 4 weeks prior to assessment. Participants were excluded if they had participated in any exercise program within 3 months of study onset or displayed evidence of significant cardiovascular, neuromuscular, endocrine, or other somatic/substance use disorders that would prevent safe participation. Five of the recruited participants failed to meet the initial eligibility criteria, and thus, a total of 60 patients were included in the final analysis.

### Sociodemographic and Clinical Information

Evaluation of all variables for each participant was performed on the same day within 2 weeks from screening date. Sociodemographic information (age, sex, years of education, duration of illness) and baseline clinical data (antipsychotic dosing, use of typical antipsychotics, fasting blood sugar [FBS], body mass index) were collected.

#### Antipsychotic Dosing

To yield antipsychotic dosing, the prescribed daily dose (PDD) in milligrams was divided by the defined daily dose (DDD) to calculate a PDD : DDD ratio. DDD is defined as the assumed average daily maintenance dose for a drug used for its main indication in adults (23).

### Psychiatric Symptoms

Psychiatric symptoms were determined *via* interviews and self-report questionnaires. Outcome measures for psychiatric symptoms included the Beck Depression Inventory (BDI), Brief Psychiatric Rating Scale (BPRS), and State and Trait Anxiety Inventory (STAI).

#### Beck Depression Inventory (BDI)

The BDI was used to assess depressive symptoms (24). The BDI is a 21-item self-report inventory that asks participants to choose the one statement that best describes their feelings during the past 2 weeks.

#### Brief Psychiatric Rating Scale (BPRS)

The BPRS is an 18-item, semi-structured introductory interview that is completed by the interviewer (25). Each item is rated on a scale from 0 to 6. Scores between 15 and 30 indicate minor symptoms, while those above 30 indicate major symptoms. The BPRS has following five subscales: Thought (items - grandiosity, hallucinations, unusual thought content, and conceptual disorganization), Withdrawal (items - disorientation, blunted affect, emotional withdrawal, and motor retardation), Anxiety and Depression (items—somatic concern, anxiety, depression, and guilt), Paranoid (items—hostility, suspicion, and uncooperativeness), and Excitement (items—tension, excitement, and mannerisms and posturing) (26).

#### State-Trait Anxiety Inventory (STAI)

The STAI is a self-rated instrument that contains two 20-item subscales for the measurement of anxiety (27). One scale measures state anxiety, while the other measures trait anxiety.

### Psychological Variables

Psychological variables included the Rosenberg Self-Esteem Scale (RSES) and World Health Organization Quality of Life Scale (QOL).

#### Rosenberg Self-Esteem Scale (RSES)

RSES is a 4-point scale consisting of 10 questions, 5 positive and 5 negative self-esteem. The higher the score, the higher the degree of positive self-esteem (28).

2.4.2 World Health Organization Quality of Life Scale Abbreviated Version (WHOQOL-BREF)

The World Health Quality Assessment Instrument-100 (29) was revised by Min et al. (30) to develop the Korean version of WHOQOL-BREF. This is composed of a total of 24 items on the 5-point scale and higher score means higher quality of life.

### Cognitive Function

#### Sternberg Working Memory (SWM) Task

The classic Sternberg Working Memory (SWM) task (31) was administered using Inquisit 5.0.13.0 (Millisecond Software, LLC, Seattle, WA, USA). Each trial of the task consisted of a set of two to five white digits presented in a sequence (1200 ms each). A yellow probe digit appeared 2500 ms after the last digit (maintenance period), at which point participants were required to press an appropriate button indicating whether the digit had been present in the previously displayed sequence. Participants were provided with visual feedback regarding the accuracy of their responses. Task sessions were divided into equally distributed “in” (probe present in the memory sequence) and “out” (probe not present in the memory sequence) trials (120 trials in total, preceded by 15 training trials).

### Functional Mobility

#### Pedometer

A pedometer is an inexpensive and easy-to-use device that measures real-time physical activity by counting an individual’s steps during daily life (19). Typical physical activity, such as walking, reflects the level of daily FM well (20), and walking has been shown to improve various aspects of FM (20). In brief, as walking is the most primary mode of physical activity, counting steps with a pedometer is useful for quantifying the level of FM (20). In the present study, The Yamax digiwalker SW-200 (Yamax Corporation, Tokyo, Japan) were used one time for two consecutive days. In order not to interfere with the daily life of research participants, we did not have any restrictions except to wear a pedometer in the waist.

#### Timed Up and Go (TUG) Test

In contrast to the pedometer, which mainly measures FM during daily living, the TUG test is a performance-based FM test that requires an individual to perform complex physical activities in a clinical setting (4, 32). The TUG test includes turning, transfers from sitting, and walking, and requires more functional movement (4). This study used the TUG test described by Podsiadlo and Richardson (22). The participants were asked to perform the TUG test at their own pace in a well-lit environment. For the TUG test, the participants were seated and were allowed to use the arms of the chair to help them stand up. During the test, the participants were observed and timed during all the time they stood up from a seated position in an arm chair, walked 3 m, turned around, walked back to the chair, and then returned to a seated position.

### Statistical Analysis

Descriptive analyses of the sociodemographic and clinical variables were performed. Quantitative variables are described as the mean ± standard deviation and qualitative variables as the number and percentage. Multiple regression analyses were used to identify predictive factors associated with FM, with adjustments for relevant covariates. After performing the correlation analyses to identify the associated factors, separate multivariate stepwise analyses were conducted, with either the TUG test or pedometer variable as the dependent variable, for FM. All analyses were performed using SPSS version 18.0 (SPSS, Chicago, IL). Statistical significance was set at p < 0.05.

## Results

### Correlation Analysis

**Table 1** shows the descriptive statistics and correlations between the clinical characteristics and FM. The Pearson correlation coefficients showed that the pedometer values was the mean number of steps per day, which were significantly positively correlated with the RSES (p = 0.033) and QOL (p = 0.047) scores, but significantly negatively correlated with the BPRS-Total (p = 0.038) and BDI (p = 0.011) scores. In addition, the TUG test times were significantly positively correlated with FBS (p = 0.031), as well as with the BPRS-Withdrawal (p = 0.008), BDI (p = 0.010), and Trait Anxiety Inventory (p = 0.026) scores; however, the TUG test times were significantly negatively correlated with the SWM correct rate (p = 0.011).

**Table 1:** Clinical characteristics and their relationships with functional mobility in people with schizophrenia (*N* = 60).

	Mean (SD) or %	Correlation with pedometer, steps/day	Correlation with Timed Up-and-Go, time (s)
Age	38.88 (10.08)	−0.15	0.25
Sex (Male)	58.30%	−0.25	0.10
Years of education	14.07 (2.96)	0.05	−0.05
Duration of illness	14.68 (10.15)	−0.20	0.10
PDD : DDD ratio	2.17 (0.98)	0.00	0.08
Typical antipsychotics use	91.66%	0.09	−0.03
FBS	106.20 (36.39)	0.01	0.30*
BMI	27.44 (5.18)	0.06	0.06
BPRS-Total	42.26 (15.94)	−0.28*	0.26
BPRS-Thought	7.04 (3.59)	−0.19	0.20
BPRS-Withdrawal	9.17 (4.02)	−0.24	0.36**
BPRS-Anxiety	7.91 (3.47)	−0.26	0.02
and Depression			
BPRS-Paranoid	5.85 (2.95)	−0.19	0.14
BPRS-Excitement	4.33 (2.14)	−0.26	0.12
BDI	17.61 (11.01)	−0.34*	0.36**
STA	45.61 (9.39)	−0.06	0.20
TRA RSES QOL SWM latency time SWM correct rate (sec)	48.63 (10.30)	−0.22 0.29* 0.27* −0.13 0.16	0.31* −0.22 −0.26 0.13 −0.35*
	21.48 (3.84)		
	45.61 (10.13)		
	2570.64 (1808.62)		
	0.63 (0.21)		

PDD, prescribed daily dose; DDD, defined daily dose; FBS, fasting blood sugar; BMI, body mass index; BPRS, Brief Psychiatric Rating Scale, BDI, Beck Depression Inventory; STA, State Anxiety Inventory; TRA, Trait Anxiety Inventory; RSES, Rosenberg Self-Esteem Scale; QOL, Quality of Life Scale; SWM, Sternberg Working Memory.

*Significant at p < 0.05 (Pearson correlation, two-tailed); **Significant at p < 0.01 (Pearson correlation, two-tailed).

### Multiple Regression Analysis

Multivariate analyses were applied to determine which variables were closely related to the pedometer values and TUG test times after adjusting for other clinical correlates. Signiﬁcantly associated variables, as determined by the univariate analyses, were included as independent variables. Separate stepwise multiple regression analyses for the pedometer and TUG test variables provided the following models. As shown in **Table 2**, after excluding the BPRS-Total, RSES, and QOL scores, the only significant predictor of the pedometer value was the BDI score, which accounted for 12% of the variance. According to **Table 3**, in the first step, the SWM correct rate, FBS, and BDI score were significant predictors of changes in the TUG test times, accounting for 36% of the variance. In the second step, the introduction of the BPRS-Withdrawal score increased this to 39%.

**Table 2:** Multivariable stepwise regression of the pedometer values (steps/day) in people with schizophrenia.

	Explanatory variables	Standardized coefficient		P	*R^2^*	F
		(ß)	t			
**Step 1**	BDI	−0.34	−2.63	0.011	0.12	6.91 (p < 0.01)
**Excluded variables**	BPRS-Total		−1.11	0.272		
	RSES		1.61	0.113		
	QOL		1.23	0.226		

BDI, Beck Depression Inventory; BPRS, Brief Psychiatric Rating Scale; RSES, Rosenberg Self-Esteem Scale; QOL, Quality of Life Scale.

**Table 3:** Multivariable stepwise regression of Timed Up-and-Go test times (sec) in people with schizophrenia.

	Explanatory Variable	Standardized Coefficient		p	*R^2^*	F
		(ß)	t			
**Step 1**	BDI	0.40	3.28	0.002	0.36	8.80 (p < 0.001)
	FBS	0.39	3.26	0.002		
	SWM correct rate	−0.28	−2.36	0.023		
**Step 2**	BDI	0.32	2.46	0.018	0.39	7.34 (p < 0.001)
	FBS	0.34	2.79	0.008		
	SWM correct rate	−0.29	−2.50	0.018		
	BPRS-Withdrawal	0.19	1.51	0.139		

BDI, Beck Depression Inventory; FBS, fasting blood sugar; SWM, Sternberg Working Memory; BPRS, Brief Psychiatric Rating Scale.

## Discussion

Previous research examining the relationship between FM and depression in patients with schizophrenia utilized tests that measured mobility during activities of daily living (e.g., pedometer, accelerometer, self-report questionnaire) (18). To our knowledge, the present study is the first to investigate the effects of depression on FM, not only during daily living with a pedometer, but also in the clinical setting using the TUG test. Additionally, we examined the effects of other disorder-related correlates on FM, including psychiatric symptoms, cognitive function, psychological status, and health-related variables. The results demonstrate that depression is the best explanatory variable for both the pedometer and TUG test values. Further, working memory and FBS were associated with the TUG test.

The present findings suggest that depression may have the most consistent influence on FM, regardless of how FM is measured, in individuals with schizophrenia. However, it is unclear what mechanisms may underlie the effects of depression on FM. Depression can affect FM owing to a combination of psychological, behavioral, and physiological factors. Patients with depressive schizophrenia are more likely to have low motivation, poor self-efficacy, psychomotor retardation, a sedentary lifestyle, and a low level of volition than are patients with non-depressive schizophrenia (17, 33, 34). Furthermore, these clinical features could result in poorer performance of up and go movements or make such patients hesitant to participate in physical activity (17). In particular, our findings of significant associations between the TUG test times and low RSES and QOL scores, which are closely related to depressive symptoms, suggest that a combination of diverse components in depression can aggravate FM. Indeed, depression was found in 34% of older people with manifest FM limitation, which suggest that both conditions may progress simultaneously (17). This shared etiology also may interfere with the functional adaptation in particular chronic schizophrenia (5, 14, 18). Nevertheless, subsequent studies are needed to reveal the complex mechanisms underlying their relationship.

Similarly, the neurological mechanisms of depression and FM in schizophrenia have not been elucidated. However, based on a study of mainly older adults, the volume of subcortical white matter hyperintensities (WMHs) increases as depressive symptoms increase, leading to FM and functional impairments (35). White matter lesions in individuals with depression are closely related to smaller caudate and putamen volumes, which can lead to impairments in movement regulation and psychomotor retardation, respectively (36, 37). Although the results are inconsistent across studies, there is some evidence supporting the presence of cerebral WMHs in patients with schizophrenia (38). Thus, patients with depressive schizophrenia can be assumed to have weighted FM limitations. In addition, WMHs in schizophrenia have been found to be associated with cognitive function (38), which may partially explain the relationship we observed between the SWM scores and TUG test times in this study.

One difference in our findings was that while the TUG test times were associated with cognition and depression, the pedometer values were only associated with depression. This is because the two measures differ in the process and the setting during the test. The pedometer is mainly used to measure mobility-related physical activity during daily living, thus motivation is an important factor (32). In contrast, the TUG test requires the rapid completion of complex physical maneuvers in an experimental setting (4). In this clinical performance-based FM test, the individual needs to process and memorize environmental information while simultaneously performing physical movements (39, 40). It also requires the planning and initiation of a series of actions (5, 39, 40). In fact, using the TUG test, Leutwyler et al. (5) showed that in patients with schizophrenia, slower processing speed, which represents cognitive dysfunction, affects FM impairments. Thus, the TUG test is a more complicated process when compared to pedometer, and in particular, the transferring and tuning components of the TUG test might tax cognition (4).

Focusing on FBS, it is well known that patients with schizophrenia are vulnerable to diabetes, impaired glucose tolerance, and metabolic syndrome (6), but the association of these factors with FM is unknown. A recent cohort study by Åström et al. (41) in the elderly demonstrated that individuals with impaired glucose regulation and diabetes show reduced FM. According to Åström et al. (41), insulin resistance can increase protein degradation and decrease protein synthesis in skeletal muscle, resulting in poor physical performance. This pathology further exacerbates glucose regulation because skeletal muscle is important for glucose uptake. In addition, hyperglycemia-induced changes in neuronal structure are most likely to contribute to the myelination of brain, and lead to reduction in neural transmission (42). Furthermore, Kumar (43) have shown diabetes is associated with greater total brain atrophy and poor motor function, independent of depression. Taken together, FBS should be considered as a significant health related factor that could affect FM in patient with schizophrenia.

Both TUG test and pedometer are valid and reliable for quantifying FM that are expected to be useful in individuals with schizophrenia (22). Since TUG test needs only a stopwatch and a chair and is easy to understand, it is ideal for patients with schizophrenia. Also, TUG test reflects complex cognitive function and physical performance, even with simplicity and cost-effectiveness (4). Thus it will be useful for intervention or evaluation to recover functional disability for the patients with schizophrenia (5). Because sedentary behavior or physical inactivity, which causes disability, are characteristics seen by patients with schizophrenia, it is necessary to accurately measure their FM in daily life. In this respect, the pedometer has the advantage of being able to overcome the limitations of subjective self-reports and can easily measure and record the mobility-related physical activity in real time (19).

To date, interventions for patients with schizophrenia have focused exclusively on psychotic symptoms such as hallucinations and delusions, with little attention being paid to depression. Considering that schizophrenia is one of the most disabling illnesses (1), a more realistic intervention goal would be for the patient to recover real-world functioning, thereby allowing for independent living and social adjustment. Our findings, at least in part, suggest the possibility that the direct treatment of patients’ depressive symptoms may improve their functional ability and quality of life by increasing their level of FM. Collectively, our results support that alleviating depression will have considerable therapeutic value for patients with schizophrenia.

There are some limitations in this study. First, although the TUG test used in this study has been used to assess FM in various clinical populations, this test has not yet been standardized for patients with schizophrenia. Second, the structured diagnostic tools were not used, but diagnostic verification was double-checked by two psychiatric practitioners. In addition, 30-item Positive and Negative Syndrome Scale (PANSS), could have provided more adequate representation of negative symptoms than BPRS. Third, since the sample size of this study was small and mainly included patients with chronic schizophrenia, our findings may not be fully generalizable to all patients with schizophrenia. Fourth, as the study was cross-sectional, no causal relationships could be established. Physical difficulties, like FM limitations, may have made patients feel more depressed. Lastly, this study did not examine the mechanisms underlying depression and FM. Nevertheless, this study demonstrated for the first time that the depression experienced by patients with schizophrenia may impair FM in both the experimental and daily-living settings based on robust and practical tests, including the TUG and pedometer assessments. Follow-up studies should evaluate the biopsychosocial mechanisms of the link between depression and FM.

## Conclusions

The present study demonstrated a strong association between depression and FM after considering other clinical correlates in individuals with schizophrenia. Depressive symptoms were correlated with FM in both the daily-living and clinical settings, as measured with a pedometer and the TUG test, respectively. Additionally, memory and FBS were found to be partially associated with the TUG test. Because patients’ actual therapeutic goal is functional recovery, improving FM by treating depression has significant clinical implications. Further studies to elucidate the mechanisms underlying the relationship between depression and FM and appropriate intervention studies to alleviate depression are needed.

## Data Availability Statement

The datasets generated for this study are available on request to the corresponding author.

## Ethics Statement

All procedures were approved by the Investigational Review Board of Severance Mental Health Hospital (IRB#: YJI130702). The patients/participants provided their written informed consent to participate in this study.

## Author Contributions

DR designed the study and structured this manuscript. J-KR wrote the protocol of exercise programs. JJ, C-HK, and DK managed the literature searches and analyses (including the statistical analysis). H-BL and S-KL contributed to the interpretation of the results. J-HS and JK wrote the first draft of the manuscript. J-HS and JK managed and contributed equally the entire this study process. All authors contributed to the article and approved the submitted version.

## Funding

This work was supported by a National Research Foundation of Korea (NRF) grant funded by the Korean government (NRF-2018R1C1B6005103) and Hallym University Research Fund (HURF-2017-61).

## Conflict of Interest

The authors declare that the research was conducted in the absence of any commercial or financial relationships that could be construed as a potential conflict of interest.
